# QcrB inhibitor Q203 (Telacebec) can synergize with clofazimine and clarithromycin to control a *Mycobacterium avium* infection

**DOI:** 10.1371/journal.pone.0344608

**Published:** 2026-04-21

**Authors:** Yong Cheng, Katie Mulvey, Garrett C. Moraski, Xuejuan Tan, Toru Mizutare, Satoshi Miyagawa, Carrie Frey, Marvin J. Miller, Jeffrey S. Schorey

**Affiliations:** 1 Department of Biochemistry and Molecular Biology, Oklahoma State University, Stillwater, Oklahoma, United States of America; 2 Department of Biological Sciences, University of Notre Dame, Notre Dame, Indiana, United States of America; 3 Department of Chemistry and Biochemistry, Montana State University, Bozeman, Montana, United States of America; 4 Shionogi and Co., Ltd., Osaki, Japan; 5 Hsiri Therapeutic Inc., Philadelphia, Pennsylvania, United States of America; 6 Department of Chemistry and Biochemistry, University of Notre Dame, Notre Dame, Indiana, United States of America; Rutgers Biomedical and Health Sciences, UNITED STATES OF AMERICA

## Abstract

The targeting of the mycobacterial electron transport chain for drug development has recently garnered significant clinical success. The cytochrome bcc complex within the electron transport chain has emerged as a viable drug target, with the antibiotic Q203 (Telacebec) showing excellent activity against *M. tuberculosis in vitro* and *in vivo*. To determine if Q203 can function as an antibiotic against the non-tuberculosis mycobacteria *M. avium* and *M. intracellulare* (MAC), MIC and bactericidal studies were performed, both against various laboratory and clinical strains of MAC as well as *in vivo* infection studies. These studies found that Q203 provides synergistic activity against all tested MAC isolates when combined with clarithromycin and provided significant added benefit in a acute *M. avium* mouse infection model when combined with clarithromycin and clofazimine.

## Introduction

The incidence of nontuberculous mycobacteria (NTM) infections has been increasing in the United States, Japan and in other countries where infections have been followed over time [1,2]. *Mycobacterium avium* complex (MAC), which consists of *M. avium* and *M. intracellulare*, is the most common NTM isolated from U.S. patients and ranges from 62 to 89% depending on geographical location [3]. MAC is an important cause of pulmonary disease in individuals with underlying lung diseases, such as cystic fibrosis and chronic obstructive pulmonary disease, and is an opportunistic pathogen in immunocompromised patients [4]. The prevalence of MAC is also increasing in individuals especially in elderly women with slender physiques with no known underlying immune deficiency [4]. MAC is ubiquitous within the environment and is found in soil, treated or untreated water, house plumbing systems, and animals [5]. MAC infections are difficult to treat and have been shown to be resistant to many of the clinically used antibiotics [6,7]. The standard of care includes involves daily or intermittent multidrug antibiotic treatment with a macrolide (clarithromycin or azithromycin), a rifamycin, and ethambutol, which continues for at least 12 months following sputum conversion (the time point at which *M. avium* is no longer detected in sputum samples). During the initial stage of treatment, significant bactericidal effects of the drugs can be very slow or delayed [8], leading to typical continuous treatment times of 18–24 months. Severe or drug resistant *M. avium* infections can require more intense treatments, including clofazimine and intravenous or aerosol amikacin or resection of parts of the infected lung [9].

Recently the respiratory chain has emerged as a potent drug target against *Mycobacterium tuberculosis*. The drug targets within the respiratory chain include the proton ATPase, which is a target for bedaquiline, a recently approved 2^nd^ line drug to treat multidrug resistant TB [10]. The cytochrome bcc complex within the electron transport chain has also emerged as a viable drug target with the drug Q203 (Telacebec) showing excellent activity against *M. tuberculosis in vitro* and *in vivo* [11,12]. In clinical trials Q203 has shown a good safety, tolerability and pharmacokinetic profile [12,13]. In contrast to *M. tuberculosis*, the potential for targeting the respiratory chain for the treatment of NTMs has not been adequately addressed. Previous study has shown that inhibitors of QcrB, which is part of the mycobacterial cytochrome bcc complex, can work against MAC when combined with clarithromycin (CLR) [14]. However, a published study with Q203 show no activity against non-tuberculosis mycobacteria (NTMs) including MAC [11,15]. However, in these studies Q203 was tested alone, no combination with any other antibiotics. To determine if Q203 is potent against MAC, especially in combination with other clinically used antibiotics, the minimum inhibitory concentration (MIC) and anti-MAC activity of Q203 in the combination with CLR and/or clofazimine in broth media was defined. The efficacy of Q203 was further investigated in a mouse MAC infection model. The studies found that Q203 provided synergistic activity against all tested MAC isolates when combined with CLR and provided significant added benefit in an acute mouse *M. avium* lung infection model when combined with CLR and clofazimine (CFZ).

## Materials and methods

### Animal

6-week-old female Balb/c (H-2d) mice were purchased from Charles River Laboratories and housed at the institutional animal facility under specific-pathogen-free conditions during the experiment. The mouse studies were approved by the Japanese Ministry of the Environment for the Humane Treatment and Management of Animals. For all mouse studies, the mice were euthanized by using isoflurane as an anesthesia followed by cervical dislocation. All mouse experiments were completed within 13 days post-treatments. Mice were observed daily and none of the mice showed signs of stress during the drug and/or infection period. Mice were euthanized when endpoint criteria were reached.

### Bacterial culture

*M. avium hominissuis* strains ATCC 700898 (MAC101) and A5 as well as all *M. intracellulare* strains were purchased from ATCC. *M. avium* serotype 4 was a kind gift form Delphi Chatterjee, Colorado State University. The Mayo Clinic, Mycology and Mycobacteriology Laboratories generously provided the de-identified *M. avium hominissuis* clinical isolates. Storage of clinical isolates in a biospecimen repository was obtained by written consent. The shipment of the samples and there use in drug screening were approved by the Mayo Clinic IRB and Biospecimens Committee. The exemption status for obtaining the clinical isolates was approved by the University of Notre Dame’s IRB (protocol number 13-09-1221). *M. avium* and *M. intracellulare* frozen stocks (stored at -80^o^C) were inoculated into bacterial culture tubes (Fisherbrand, Cat.14-956-1J) containing 5 mL of Middlebrook 7H9 medium (plus 10% OADC) as described previously [14]. The *M. avium* were grown until exponential phase (OD600 = 0.8–1.5) in a bacterial shaker at a speed of 150 rpm at 37^o^C for 3–5 days before use. The McFarland standard was used to calculate the mycobacteria concentration in broth cultures.

### MIC assay

The *M. avium* or *M. intracellulare* cultures, grown in 7H9 medium plus 10% OADC (Oleic acid, Albumin, Dextrose, Catalase), were diluted to a final concentration of 1.0 X 10^6^ CFU/mL. Compounds (originally dissolved in DMSO) were serially diluted in 7H9 medium plus 10% OADC and 100 μl was added to each well of a 96-well plate to give the indicated concentrations. Highest concentration of Q203 tested was 10 μg/ml and 2-fold serially dilutions were used, with the lowest concentrations equal to 0.0078 μg/ml. MICs for CLR was defined for each MAC strain and all Q203 concentrations were tested using clarithromycin concentrations of 0.0625 μg/ml or 0.125 μg/ml for *M. avium* and 0.0078 μg/ml, 0.0312 μg/ml and 0.125 μg/ml for *M. intracellulare*. 100 µL of the diluted *M. avium* or *M. intracellulare* culture was added into each well of a 96-well plate and mixed gently. The final volume per well was 200 μl. The plates were wrapped with aluminum foil and incubated at 37^o^C for 3 days. 30 µL of resazurin solution (0.01%, in distilled water, sterilized through 0.2 μm filter) was added into each well and plates were incubated at 37^o^C overnight (16–24 hr) [14]. A conversion in color from blue to pink indicates metabolic activity of MAC while remaining blue indicates no metabolic activity. Each well was visually scored as either blue or pink and the concentration of drug or drug combinations where no change from blue to pink was used to define the MIC. FIC index calculation: (Q203 MIC in combination/Q203 MIC alone) + (CLR MIC in combination/CLR MIC alone). A FIC index of ≤ 0.5 was defined as synergistic, > 0.5 and ≤ 1.0 as additive effect, > 1.0 and ≤ 4.0 as indifference, and > 4 as antagonistic effect. When the MIC for Q203 alone was above 10 μg/ml, the Q203 MIC concentration used in the FIC index calculation was set to 10 μg/ml.

### *M. avium* Killing assay

*M. avium* strain MAC101 was grown in Middlebrook 7H9 media plus 10% OADC, and cultured in a bacterial shaker at 37˚C until mid-exponential phase as described above. Mycobacterial killing assay in bacterial broth was performed in 96-well plates as previously described [14]. The following drug combinations were used in the test: Single drug treatment: Q203 (0, 1, or 10 µg/ml); Dual drug treatment Q203 (0, 1, or 10 µg/ml) + CLR (0.1, 0.5, or 1 µg/ml); Triple drug treatment Q203 (0, 1, or 10 µg/ml) + CLR (0.1, 0.5, or 1 µg/ml) + CFZ (0.1 µg/ml). Wells were adjusted to a total volume of 200µl using Middlebrook 7H9 media plus 10% OADC. The plates were sealed with parafilm and incubated for 7 days at 37˚C + 5% CO_2_. Each well was serially diluted in phosphate-buffered saline (PBS) and spread onto 7H10 agar plates plus 10% OADC. Plates were incubated at 37˚C until mycobacterial colonies were visible. Mycobacterial colonies were counted to define the number of remaining viable bacteria. The results were analyzed using the unpaired Student’s *t*-test with all the data compared to no Q203 (i.e., vehicle only, CLR no Q203 or CLR + CFZ no Q203). Significance was defined at a P value of ≤0.05.

### Pharmacokinetics of Q203 in rat and mouse

In the rat PK study, male Sprague−Dawley rats (8 weeks) were used. Compounds were formulated as solutions in dimethyl sulfoxide /0.5% methylcellulose, 400cP (1:4, 0.4 μmol /mL) and dosed orally at 2 μmol/kg (n = 2) in nonfasted condition. Blood samples (0.2 mL) were collected with 1-mL syringes containing anticoagulants (EDTA-2K and heparin) at 0.5, 1, 2, 4, 6, 8, and 24 h after dosing. Compounds were formulated as solutions in dimethyl sulfoxide /propylene glycol (1:1, 1 μmol /mL) and dosed intravenously from the tail vein at 1 μmol /kg (n = 2) under isoﬂurane anesthesia and the nonfasted condition. Blood samples (0.2 mL) were collected with 1-mL syringes containing anticoagulants (EDTA-2K and heparin) at 3, 10, 30, 60,120, 240, and 360 min after dosing. Blood samples were centrifuged to obtain plasma samples, which were transferred to each tube and stored in a freezer until analysis. Plasma concentrations were determined by LC/MS/MS. Pharmacokinetic parameters were calculated using WinNonlin based on a non-compartment model.

In the mouse PK study, female BALB/c mice (8 weeks) were used. Compounds were formulated as solutions in tween20/mixture of 20% hydroxypropyl-β-cyclodextrin and 0.6% hydroxypropyl methylcellulose at pH 3 (1:4, 1 mg/mL) and dosed orally at 1 mg/kg (n = 3) in nonfasted condition. Blood samples (0.03 mL) were collected with capillary containing anticoagulants (EDTA-2K and heparin) at 1, 2, 4, 8, and 24 h after dosing. Blood samples were centrifuged to obtain plasma samples, which were transferred to each tube and stored in a freezer until analysis. Plasma concentrations were determined by LC/MS/MS. Pharmacokinetic parameters were calculated using WinNonlin based on a non-compartment model.

### Assessment of Q203 efficacy in mice

7-week-old female Balb/c mice were infected with 3.4 x10^6^ CFU (colony forming units) of *M. avium* MAC101 strain by intranasal infection in a total volume of 70μl [14]. Four mice from each batch of *M. avium* infections were humanely sacrificed 1 day after infection to determine the level of mycobacteria in the lungs at the starting point of drug treatment. The drug treatment was initiated 1-day post-infection and administered by oral gavage once per day for 5 days. In a separate study, mice were infected with 2.6 x 10^6^ CFU by intranasal infection. Four mice from each batch of *M. avium* infections were humanely sacrificed 1 day after infection to determine the level of mycobacteria in the lungs at the starting point of drug treatment. The drug treatment was initiated 1-day post-infection and administered by oral gavage once per day for 5 days followed by 2-day rest and additional treatment for 5 days. The mice were observed daily and none of the mice showed signs of stress during the infection period and all mice were humanely euthanized at the end of the study period. The drug doses were as follow: Q203 (5 mg/kg), CLR (200 mg/kg), CFZ (20 mg/kg), rifampin (20 mg/kg) and ethambutol (100 mg/kg). Except for Q203, the concentration of drugs was based on previously published MAC mouse infections studies [16,17]. The Q203 concentration was based on previous mouse infection studies with *M. ulcerans* [18,19]. For the vehicle control groups, mice were treated by oral gavage with the drug solvent (20% Tween 20, 80% aq. (20% 2-hydroxypropyl)-β-cyclodextrin and 0.6% hydroxypropyl methylcellulose, pH 3).

All mice were sacrificed 1 day after the final dosing and the lung homogenate was prepared in phosphate-buffered saline (PBS) containing 0.05% (vol/vol) of Tween 80. The tissue homogenate was appropriately diluted in the same buffer, and 50 µl of the diluted homogenate was spread on Middlebrook 7H11 agar plates with 10% OADC, 0.5% glycerol and 0.05% Tween 80, and containing a cocktail of fungizone and PANTA (polymixin B, amphotericin B, nalidixic acid, trimethoprim, and azlocillin). *M. avium* colonies were counted after 10–14 days of incubation at 37^o^C and expressed as log_10_ CFU per organ. The results were analyzed using the unpaired Student’s *t*-test with all the data compared to vehicle control except for the CLR + CFZ + Q203 which was also compared to the CLR + CFZ group. Significance was defined at a P value of ≤0.05.

## Results

### In vitro efficacy of Q203 against laboratory and clinical isolates of MAC

Q203 was initially tested alone or in combination with CLR against both laboratory strains and clinical isolates. The MIC for Q203 alone was ≥ 10 μg/mL for most tested *M. avium* strains (Table 1). However, a 0.0078 μg/mL MIC for Q203 was observed for most clinical isolates in the presence of 0.125 μg/mL of CLR. Interestingly, the laboratory strains were more resistant to CLR + Q203 than the clinical isolates. At the CLR concentration of 0.0625 μg/ml, all the laboratory strains showed MICs of >10 for Q203 while at the same CLR concentration the clinical isolates showed MICs for Q203 between 0.25 and 0.0078 μg/ml. Since for many strains the MIC for Q203 alone was not observed at the highest concentration tested (10 μg/ml) due to issues with drug solubility we used the highest concentration tested for our FIC calculations. The use of 10 μg/ml as the MIC results in an underestimation of the number of strains that show synergy between Q203 and CLR. Nevertheless, for at least one CLR concentration, both the laboratory and clinical isolates showed FIC index scores of ~0.5 or less indicating synergy between CLR and Q203. We also tested 5 ATCC strains of *M. intracellulare* and again observed synergy between Q203 and CLR (Table 2).

**Table 1:** MIC for Q203 against *M. avium* strains.

*M. avium* Strains	MIC (Q203, μg/ml)			MIC (CLR only, μg/ml)	FIC Index (Q203 + CLR)	
	CLR (0 μg/ml)	CLR (0.0625 μg/ml)	CLR (0.125 μg/ml)		CLR (0.0625 μg/ml)	CLR (0.125 μg/ml)
MAC 101	> 10	> 10	0.0156	0.25	N/A	0.50156
A5	> 10	> 10	0.25	0.25	N/A	0.525
Serotype 4	> 10	> 10	0.0156	1	N/A	0.12656
Clinical Isolate #7	> 10	0.0078	–	0.125	0.50078	N/A
Clinical Isolate #22	> 10	0.25	0.125	0.25	0.275	0.5125
Clinical Isolate #28	0.0312	0.0156	0.0078	1	0.5625	0.375
Clinical Isolate #47	0.25	0.0078	0.0078	1	0.0937	0.1562
Clinical Isolate #50	10	0.125	0.0078	1	0.075	0.12578
Clinical Isolate #58	> 10	0.0078	0.0078	0.25	0.25078	0.50078
Clinical Isolate #61	> 10	0.0312	0.0078	1	0.06562	0.12578

**Table 2:** MIC for Q203 against *M. intracellulare* strains.

*M. intracellulare* Strains	MIC (Q203, μg/ml)				MIC (CLR only, μg/ml)	FIC Index (Q203 + CLR)		
	CLR (0 μg/ml)	CLR (0.0078 μg/ml)	CLR (0.0312 μg/ml)	CLR (0.125 μg/ml)		CLR (0.0078 μg/ml)	CLR (0.0312 μg/ml)	CLR (0.125 μg/ml)
ATCC # 700662	< 0.0078	< 0.0078	< 0.0078	< 0.0078	> 0.125	N/A	N/A	N/A
ATCC # 35762	> 10	> 10	0.0625	-	0.125	N/A	0.25585	N/A
ATCC # 35769	> 10	> 10	< 0.0078	-	0.125	N/A	0.25038	N/A
ATCC # 35848	> 10	0.0312	< 0.0078	-	0.125	0.06552	0.25038	N/A
ATCC # 35763	> 10	> 10	0.0312	-	0.125	N/A	0.25272	N/A

**Fig 1:**
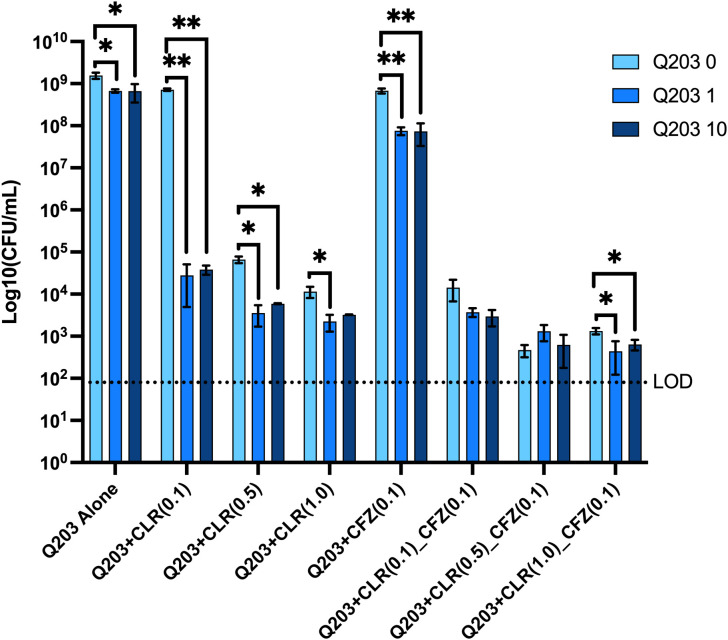
Q203 can synergize with clarithromycin to kill *M. avium.* MAC101 was incubated in the presence or absence of drugs for 7 days. Remaining bacteria were quantified by serial dilution of individual wells and plating on Middlebrooks 7H10 agar plates. Data are mean ± SD (Standard deviation) from triplicate wells. LOD, limit of detection. Compared to the vehicle control; * p < 0.05 and ** p < 0.005 by unpaired Student’s *t*-tes*t*. Representative of two experiments.

Since the resazurin assay, used in the MIC studies, measures metabolic activity, it cannot be used to measure bactericidal activity. Therefore, a direct quantification of the number of bacteria following a 7-day treatment with Q203 alone or in combination with CLR and CFZ was performed. Although the MIC assay did not show any effect of Q203 alone, a slight decrease in ATCC 700898 strain (MAC101) growth with the addition of Q203 compared to untreated controls was observed. CLR, at 0.1 μg/mL also showed minimal decease in CFU compared to untreated MAC101; however, when CLR was combined with 1 or 10 μg/mL Q203 a 4-log reduction in CFU was observed (Fig 1). Since the inoculum was 1.64 x 10^7^ CFUs at the time drug treatment was initiated, the combination of 0.1 μg/mL CLR + Q203 clearly showed bactericidal activity. 0.5 μg/mL CLR was also bactericidal as we observed a 4-log drop in CFU compared to untreated control; with another ~1 log CFU drop when combined with 1 or 10 μg/mL Q203 (Fig 1 and S1 Table). Previous studies have shown that CFZ can substitute for rifampicin in treatment of *M. avium* pulmonary disease [20]. Numerous published studies have also shown that CFZ can be effective against MAC and other NTMs [21–25]. To evaluate if Q203 can provided added benefit to the combination of CFZ and CLR, a double and triple drug combination experiments and evaluated the bacterial counts after 7 days of drug treatment was performed. The presence of 0.1 μg/mL of CFZ had limited effect on bacterial growth; however, the addition of 1 or 10 μg/mL of Q203 resulted in a 1 log decrease in CFU compared to CFZ alone (Fig 1). The combination of 0.1 μg/mL of both CFZ and CLR resulted in a significant (~4.5 log) decrease in CFU compared to no drug treatment (Fig 1 and S1 Table). The addition of Q203 to CFZ and CLR resulted in an additional 0.5 log decrease in CFU, although the decrease was not statistically significant. No consistent effect of Q203 was observed with higher concentrations of CLR when combined with 0.1 μg/mL of CFZ.

### *In vivo* efficacy of Q203 against *M. avium* in a mouse infection model

We first evaluated the pharmacokinetics of Q203 in rats. Similar to previous studies [26] a CL of 5.2 mL/min/kg, a Vdss of 3.9 L/kg, a T_½ of 11 h, and a BA of 74% was observed. Using a 1 mg/kg dose a Cmax of 155 + /- 13 ng/mL, AUC of 2203 + /- 77 ng per h/mL and a Tmax at 3.0 + /-1.2 h was defined (Table 3).

**Table 3:** PK values for Q203.

ND-011496	Q203
MAC+0.125CLR nM	6.7
microsome_h/r_%	30/78
Hep_120min_h/r_%	77/84
CYP	All >21
MBI	Negative
Solubility at pH 1.2/pH6.8	-/-
IV AUC (0 - inf) nM*hr	3,300
PO AUC (0 – inf) nM*hr	4860
F%	0.87
Cmax nM	295
Tmax h	4
CLtot_mL/min/kg	5.2
T_1/2_h	11
BA_%	74
Vdss_L/kg. Kp_Liver/Lung	3.9 18/5.1
hERG@3mM_%	>30mM
FAT	Negative

**Fig 2:**
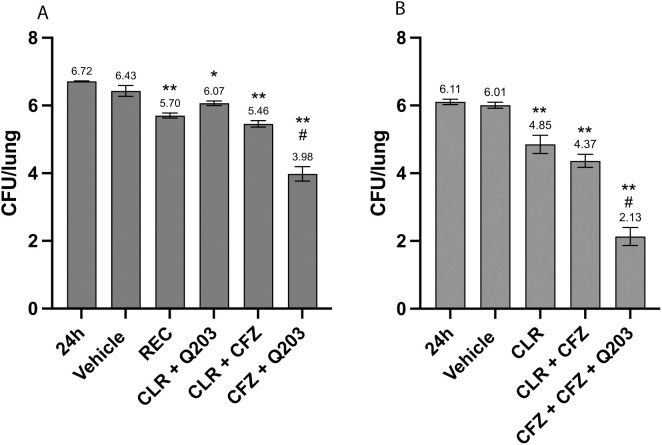
Q203 potentiates the antimicrobial activity of CLR and CFZ in a *M. avium* mouse infection model. MAC101-infected mice were treated with the indicated combination of antibiotics 1 day post-infection for a period of 5 days (A) or 10 days **(B)**, and the lungs were harvested 1 day after final drug treatment. Lung homogenates were serial diluted and plated to define bacterial numbers (CFU). Data are mean ± SD (Standard deviation) from 4 mice per group. Compared to vehicle control; * p < 0.05 and ** p < 0.005 by unpaired Student’s *t*-tes*t*. Compared to the CLR + CFZ group; ^#^ p < 0.001 unpaired Student’s *t*-test.

To determine if Q203 can effectively control an MAC lung infection *in vivo,* an acute infection model using the well-characterized MAC101 strain was performed. This entailed infecting mice with approximately 10^6^ CFU intranasally and initiating antibiotic treatment 1-day post-infection. No effect of Q203 + CLR in controlling a MAC101 infection *in vivo* was observed after 5 days of drug treatment (Fig 2A and S2 Table). In contrast, mice treated for 5 days with 200 mg/kg CLR + 20 mg/kg CFZ resulted in an ~ 1.0 log decrease in lung CFUs compared to vehicle control. The combination of 200 mg/kg CLR + 100 mg/kg ethambutol + 20 mg/kg rifampin (REC), which is the standard-of-care antibiotic combination used for drug sensitive *M. avium* infections [27], showed a similar effectiveness, reducing CFU in the lung by ~0.7 log relative to vehicle treated mice. The addition of 5 mg/kg of Q203 to the CLR + CFZ resulted in a highly significant decrease in lung CFU, with a 1.5 log drop relative to CLR + CFZ and 2.7 log drop compared to vehicle control (Fig 2A and S2 Table). This difference was even more striking when the treatment time was increased to 10 days with a 4 log drop in lung CFU compared to untreated infected mice and 2.2 log decrease in CFU compared to CLR + CFZ (Fig 2B and S2 Table). Together, the data shows that Q203 is highly effective, when combined with CLR and CFZ in controlling MAC101 in a mouse acute infection model.

## Discussion

With the increased incidence of MAC infections worldwide and the limited options for effective antibiotic treatment, there is a clear need to identify new drug targets and new antibiotics that target them. Recently, drugs targeting the cytochrome bcc-aa3 complex in *M. tuberculosis* have shown promise in laboratory models and human studies [28,29]. In this study Q203 was tested as an inhibitor of QcrB, a protein within the mycobacterial cytochrome bcc-aa_3_ complex. Its activity was found not to be limited to *M. tuberculosis*, as it showed synergistic activity against multiple laboratory and clinical isolates of MAC when combined with clarithromycin. Like previous studies, [15] a limited activity of Q203 alone against most of the tested MAC and *M. intracellulare* strains was observed (Tables 1, 2). The reason for the resistance is not known but it may be due to the fact that *M. avium* and Mtb express two terminal oxidases and Q203 is specific for cytochrome bc1-aa3 and has no activity against the cytochrome bd oxidase, suggesting redundancy [30]. This hypothesis is supported by the observation that *M. ulcerans* only express the cytochrome bc1-aa3 and is highly susceptible to Q203 [31]. In contrast, *M. abscessus* has a naturally-occurring polymorphisms in QcrB, which is responsible for its resistance to Q203 [32]. This polymorphism is not observed in MAC.

However, Q203 was shown to synergize with clarithromycin, which is noteworthy as it is a staple antibiotic for the treatment of drug-sensitive MAC infections [9]. Previous studies conducted with Q203 have shown it to be highly active against *M. tuberculosis* H37Rv in a macrophage infection model [33] and to synergize with other anti-TB drugs, including Macozinone (PBTZ169) [34], vancomycin, and rifampicin [35] in a mouse TB infection model. To our knowledge, this study is the first to show synergistic activity between Q203, CLR and CFZ in MAC infected mice. The mechanism for this synergism is unclear but may be due to Q203’s known ability to reduce the expression of proteins required for the synthesis of the cell wall lipids phthiocerol dimycocerosates and phenolic glycolipids [35].

The clear benefit of adding CFZ to the combination of CLR and Q203 was initially unexpected as Q203 was not shown to potentiate the activity of CFZ against *M. abscessus* [36]. However, this is likely due to naturally occurring polymorphisms in the *M. abscessus* QcrB gene, rendering the protein resistant to Q203 activity [32]. CFZ was originally developed in the 1950s as a TB antibiotic but the results in various animal studies were inconsistent and its use for treatment of TB was not pursued [37]. Later it was repurposed for treating leprosy and remains part of the standard-of-care for leprosy [38]. In the 1980s, CFZ was evaluated for the treatment of NTMs and was found to be effective against various species of NTMs including MAC and *M. abscessus*. However, due to drug access issues and the skin discoloration associated with CFZ use, it is recommended that CFZ be used as an alternative drug against difficult to treat NTM infections [39]. Nevertheless, it is clear from clinical studies that CFZ is a viable antibiotic for treatment of MAC, and that in combination with other antibiotics, could be used as part of a first-line drug regimen.

At present, it is unclear why such significantly enhanced efficacy was observed when CFZ was added to a CLR + Q203 regimen in the mouse infection model. CFZ appears to have multiple modes of action against mycobacteria including inducing the production of reactive oxygen species, superoxide and H_2_O_2_, [40]. Disruption of the membrane structure and function has also been proposed as the mechanism of its antimicrobial activity against mycobacteria [41]. A more recent theory, suggests that clofazimine interacts with bacterial membrane phospholipids to generate antimicrobial lysophospholipids [42]. The bactericidal efficacy of CFZ results from the combined membrane-destabilizing effects of both clofazimine and lysophospholipids, which interfere with the K^+^ uptake and, ultimately, the ATP production. It may be the effect on ATP production that is responsible for synergistic effect of CFZ and Q203; however, additional studies are needed to test this hypothesis. In addition to the potentiating effect of CFZ, there may be other antibiotics that can function in combination with Q203. Previous studies with the efflux inhibitor verapamil, as well as other efflux inhibitors, have shown to increase Q203 potency against Mtb [43]. Efflux inhibitors should also be tested in combination with Q203 to determine if similar increased potency is observed with MAC.

The data suggest that Q203 has the potential to be repurposed for treating MAC infections and that the cytochrome bcc-aa_3_ complex is a viable target for antibiotic development. The mouse studies suggest that Q203, CFZ and CLR may show increased efficacy compared to the present treatment standard. However, it is important to note that the in vivo studies were from acute infections and further studies of Q203 should involve testing it in a chronic mouse infection model, which is more analogous to what is observed clinically. *In vivo* studies should also be done to define the activity of the triple drug combination against other strains of MAC, including clinical isolates that are CLR resistant, which is commonly observed clinically. Development and testing of additional QcrB inhibitors should also be a priority.

## Supporting information

S1 Table Raw data for Fig. 1. (XLSX)

S2 Table Raw data for Fig. 2. (DOCX)
