# Supplementary material for: QcrB inhibitor Q203 (Telacebec) can synergize with clofazimine and clarithromycin to control a Mycobacterium avium infection
Source: PLoS One. 2026 Apr 21;21(4):e0344608. doi: 10.1371/journal.pone.0344608 (PMC13098918; doi:10.1371/journal.pone.0344608)
Supplement: S2 Table — (DOCX) [file pone.0344608.s002.docx]

Figure 2A: CFU data for individual mice

Figure 2B: CFU data for individual mice
